# Iminoflavones Combat 1,2-Dimethyl Hydrazine-Induced Aberrant Crypt Foci Development in Colon Cancer

**DOI:** 10.1155/2014/569130

**Published:** 2014-06-05

**Authors:** V. Ganga Prasad, Shishir Kawade, B. S. Jayashree, Neetinkumar D. Reddy, Albi Francis, Pawan G. Nayak, Anoop Kishore, K. Nandakumar, C. Mallikarjuna Rao, Rekha R. Shenoy

**Affiliations:** ^1^Department of Pharmacology, Manipal College of Pharmaceutical Sciences, Manipal University, Manipal, Karnataka 576104, India; ^2^Department of Pharmaceutical Chemistry, Manipal College of Pharmaceutical Sciences, Manipal University, Manipal, Karnataka 576104, India

## Abstract

The aim of the present study was to evaluate the antitumor potential of iminoflavones in *in vitro* and *in vivo* anticancer models. Preliminary screening in various cancer cell lines revealed four potential iminoflavones out of which IMF-8 was taken based on its activity against colon cancer cells. This was further confirmed by observing the nuclear changes in the cells by AO/EB and Hoechst 33342 staining studies. *In vivo* activity was assessed by dimethyl hydrazine-(DMH-) induced colon cancer model in rats. Animals were administered DMH (20 mg/kg, b.w. for 10 weeks and 30 mg/kg b.w., *i.p.* for 10 weeks) and were supplemented with (IMF-8) iminoflavone-8 (200 mg/kg, *p.o.* for 14 days). Results showed that DMH induced 100% aberrant crypt foci (ACF) and polyps which were significantly reduced in the IMF-8 treated group. IMF-8 significantly increased the catalase and GSH levels whereas it reduced the TNF-**α** and IL-6 levels markedly which suggests the antioxidative and anti-inflammatory actions of flavonoids present in IMF-8. The histopathological images of the IMF-8 treated colon showed no signs of mucosal crypt abscess. These findings suggest that the semi-synthetic iminoflavones, IMF-8, effectively inhibit DMH-induced ACFs and colonic crypts by alleviating the oxidative stress and suppressing the inflammation.

## 1. Introduction

Dietary flavonoids are commonly found in various items of plant origin.* In vitro*, flavonoids are proven to exhibit antiproliferative action for a range of cancer cell lines [1]. Contemporary research has reported potent anticarcinogenic actions of flavonoids in treatment of colon cancer [2]. Today, colon cancer is the most commonly diagnosed cancer and the second leading cause of death in the USA [3]. The global prevalence of colon cancer points to effective treatment strategies for the prevention and treatment of colon cancers. Approximately, 60% of the drugs that are being used currently are natural products [4].

Huge emphasis is being given to natural products for their anticancer properties owing to their health benefits and few side effects and drawbacks of chemotherapeutic agents. Phenolic compounds have been shown to possess antioxidant activity [5] based on their hydroxyl group. Moreover, phenolic compounds also possess a wide spectrum of biological activities such as antimutagenic [6], anticarcinogenic [7], anti-inflammatory [8], and antiallergic activities [9], as well as the ability to modify gene expression [10]. Importantly, a flavonoid derivative, flavopiridol, was widely used in traditional medicine and was a novel semisynthetic flavone, from Indian tree. We synthesized a series of novel substituted iminoflavones (IMF) and reported its cytotoxic activity [11]. Out of the 12 synthesized derivatives, IMF-2 [4-{4-[2,4 Dinitro-phenyl-hydrazine]-4H-chromen-2yl}-benzonitrile], IMF-4 [N-[2-(4-Bromo-phenyl)-chromen-4ylidene]-N′-(2,4 dinitro-phenyl)-hydrazine], IMF-5 [N-(2,4 Dinitro-phenyl)-N′-(2-pyridine-4yl-chromen-4ylidine)-hydrazine], and IMF-8 [(4-{4-[2,4 Dinitro-phenyl-hydrazono]-4H-chromen-2yl}-phenyl)-dimethyl-amine] were found to possess a low IC_50_ against Hela and HepG2 cell lines. However, iminoflavones had not been tested experimentally for their influence on colon cancer. IMF-2, -4, -5, and -8 were screened against human colon cancer cell line (HCT-116). Hence, we selected an iminoflavone, IMF-8, from our pilot studies to assess its potential on dimethyl hydrazine-induced colon cancer in albino rats of Wistar strain.

## 2. Materials and Methods

### 2.1. Chemicals

Dimethyl hydrazine, MTT, and SRB were procured from Sigma-Aldrich Co. LLC, St. Louis, MO, USA. 5-Fluorouracil was purchased from Biochem Pharmaceutical Industries Ltd., Mumbai, Maharashtra, India. MCF-7, MDA-MB-231, EAC, and HCT-116 cell lines were procured from National Centre for Cell Science (NCCS), Pune, Maharashtra, India. Media, serum, and cell culture products were procured from Invitrogen Bio Services India Pvt. Ltd., Bangalore Karnataka, India.

### 2.2. Synthesis of Iminoflavones

A series of novel substituted iminoflavones were assessed which were designed and synthesized previously in our laboratory [11]. In brief, the general scheme of synthesis for iminoflavones involves the following steps. Synthesis of chalcones involves Claisen-Schmidt condensation, synthesis of flavones involves cyclisation of chalcones, and synthesis of iminoflavones involves condensation of 2,4-DNP with flavone moiety.

### 2.3. Preliminary* In Vitro* Cytotoxicity Studies

Sulforhodamine-B and Trypan blue exclusion assays [12, 13] were performed using HCT-116, MCF-7, MDA-MB-231, and EAC cell lines. Similarly, AO/EB and Hoechst 33342 staining [14, 15] were done to examine the nuclear morphological changes within the cells. SRB assay which measures total protein content of the cells is directly proportional to viable cell count. Trypan blue dye exclusion assay is a well-accepted direct method for the measurement of cell death. Staining the cells with fluorescent dyes, including acridine orange and ethidium bromide, is used in evaluating the nuclear morphology of dead cells. To confirm whether apoptosis or necrosis has been induced by selected iminoflavones, colon cancerous cells (HCT-116) were analyzed in the presence of acridine orange and ethidium bromide staining (AO/EB) staining. As a control, untreated cells were cultured in complete media and stained with AO/EB.

The apoptotic index (AI) was calculated to confirm that iminoflavones caused cell death via apoptosis. AI is described as the percentage of apoptotic cells and apoptotic bodies within the overall population of cells. An apoptotic index was determined as the percentage of apoptotic cells, at least 200 counted cells under observation using an inverted microscope.

### 2.4. Acute Toxicity Studies

The acute oral toxicity study was conducted as per the OECD test guideline 420 (acute oral toxicity, fixed dose procedure) [16, 17]. Twelve Swiss albino mice weighing 22–25 G (11-12 weeks old) were used for the toxicity studies. Test compounds were orally administered with a single dose of 300, 1000, and 2000 mg/kg b.w. Animals were observed for possible behavioural changes such as tremors, convulsions, sleep, altered feeding, salivation, altered somatomotor activities, and diarrhoea. These observations were continued for a period of 14 days, following which animals were sacrificed to examine gross changes to the vital organs.

### 2.5. Animals and Treatments

Male Wistar rats inbred at Central Animal Research Facility, Manipal University, Manipal, India, were used in the study. Animals were acclimatized to the experimental room having temperature 23 ± 3°C, controlled humidity conditions 75%, and 12 : 12 hours light and dark cycle. Rats were housed in sterile polypropylene cages containing sterile paddy husk as bedding material. The animals were fed on autoclaved rat feed and water. Eight to ten weeks old male rats weighing 120 ± 10 g were used for the study. The animal care and handling was carried out in accordance with guidelines issued by Institutional Animal Ethics Committee, Manipal University, Manipal, Karnataka, India (number IAEC/KMC/89/2012). Animals were divided into four groups (*n* = 6), namely, normal control, DMH control, 5-fluorouracil, and IMF-8. The normal control received 0.25% CMC,* p.o.* Animals were administered DMH weekly once at a dose of 20 mg/kg body weight (*i.p.*) for 10 weeks and 30 mg/kg b.w.,* i.p.*, for 10 weeks. 5-FU was given at a dose of 10 mg/kg,* i.p.*, and IMF-8 at 200 mg/kg,* p.o*. One animal from each group was sacrificed and examined for aberrant crypt foci (ACFs), polyps, adenoma, and adenocarcinoma after 20 weeks. After confirming the induction of colonic ACF (methylene blue staining), animals were randomized according to body weight and treatments started.

### 2.6. Parameters Monitored in DMH Model

At Day 15, the animals were sacrificed and colons and livers were dissected out and perfused with an ice cold saline transcardially. The tissues were blot-dried and weighed and 10% of homogenate was prepared with ice cold (150 mM) potassium chloride solution using a homogenizer (RQT-124A/D, REMI group, Mumbai, Maharashtra, India). The homogenate was used for the following estimations, namely, GSH [18], catalase [19], nitrite, TNF-*α*, and IL-6 [20]. In the DMH model, ACF incidence, polyps and ACF count, colon length/weight ratio, and histopathological examinations were performed.

### 2.7. Statistical Analysis

Data were analyzed by one-way analysis of variance (ANOVA) and significant differences amongst treatment groups were evaluated by post hoc Tukey's test. The results were considered significant at *P* < 0.05. All statistical analyses were done using Prism 5.03 Demo Version (GraphPad Software Inc., La Jolla, CA, USA).

## 3. Results

### 3.1. Preliminary* In Vitro* Cytotoxicity Studies

Based on preliminary screening by SRB assay in HCT-116, MCF-7, MDA-MB-231, and EAC cell lines, IMF-8 [dimethylamino substituted iminoflavone] was selected for further studies (Table 1). In HCT-116 cells, IMF-8 showed an IC_50_ of 2.78 ± 0.10 *μ*M in the SRB assay. Similarly, IMF-8 was treated with EAC cells for 3 h to evaluate number of dead cells. IMF-8 was found to have an IC_50_ of 36.6 ± 3.2 *μ*M in the Trypan blue exclusion assay. Doxorubicin and IMF-8 showed apoptotic indices of 46 ± 1.73 and 33.3 ± 3.76, respectively. IMF-8 has comparable activity with standard drug doxorubicin when assessed by the AO/EB staining. Likewise, by the Hoechst 33342 staining, doxorubicin and IMF-8 showed apoptotic indices of 38.7 ± 2.40 and 34.7 ± 2.96, respectively (Table 2).

### 3.2. Acute Toxicity Studies

All the mice were alive and healthy for 14 days. IMF-8 was found to be safe up to 2000 mg/kg. Hence, the dose selected was one tenth of the safe dose, that is, 200 mg/kg.

### 3.3. Parameters Monitored in DMH Model

100% ACF incidence and polyps were seen in all the treatment groups except in normal control. They were found to be reduced in 5-FU and IMF-8 in comparison with DMH control. In DMH control, 5-FU, and IMF-8, ACF count was found to be 16.85 ± 1.5, 11.6 ± 1.0, and 12.5 ± 1.6, respectively (Table 3). Treatments with 5-FU and IMF-8 showed (*P* < 0.001) significant reduction in ACF formation as compared to DMH control. The colon length/weight ratio was found to be significantly reduced in DMH control as compared to normal control. In all the treated groups, colon length/weight ratio was increased but not significant with DMH control except 5-FU treatment (data not shown). In all treatment groups there were no significant differences in liver and spleen index as compared to normal and DMH control.

### 3.4. Biochemical Investigations

**Figure 1:**
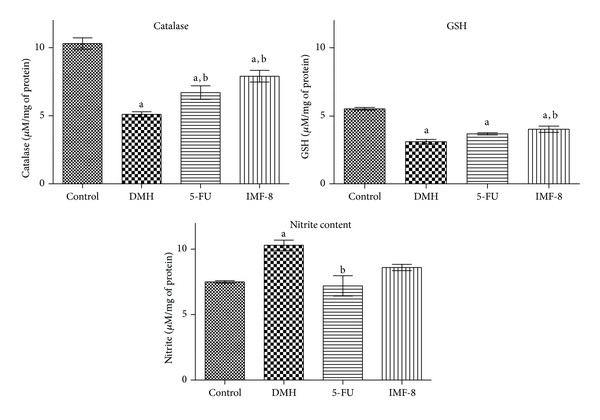
Effects of IMF-8 on the catalase, glutathione (GSH) and nitrite levels in normal control, 5-FU and DMH control in albino rats. ^a^ *P* < 0.05 when compared with control; ^b^ *P* < 0.05 when compared with DMH. Data is expressed as Mean ± SEM where *n* = 6.

DMH control showed a significant decrease in endogenous antioxidants, namely, catalase, GSH, and a rise in nitrite content as compared to normal control. 5-FU and IMF-8 showed a significant increase in the catalase activity as compared to DMH control. Only IMF-8 treatment was able to maintain high GSH level as compared to DMH control. IMF-8 was able to decrease nitrite content but not significant with DMH control, whereas 5-FU showed a significant decrease in nitrite content (Figure 1).

**Figure 2:**
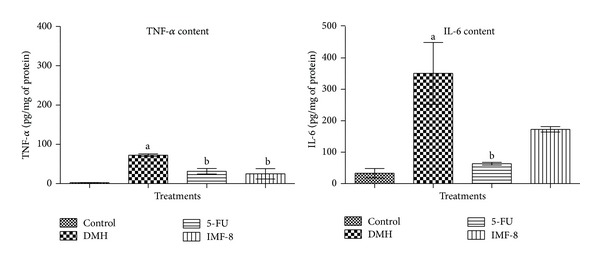
Effects of IMF-8 on the TNF-*α* and IL-6 levels in normal control, 5-FU and DMH control in albino rats. ^a^ *P* < 0.05 when compared with control; ^b^ *P* < 0.05 when compared with DMH. Data is expressed as Mean ± SEM where *n* = 6.

A significant rise in TNF-*α* and IL-6 levels was noted in DMH control compared to normal control. The 5-FU and IMF-8 treatments were able to decrease TNF-*α* level. There was no significant decrease in IL-6 levels in all the treatment groups except 5-FU standard (Figure 2).

### 3.5. Histopathological Results

**Figure 3:**
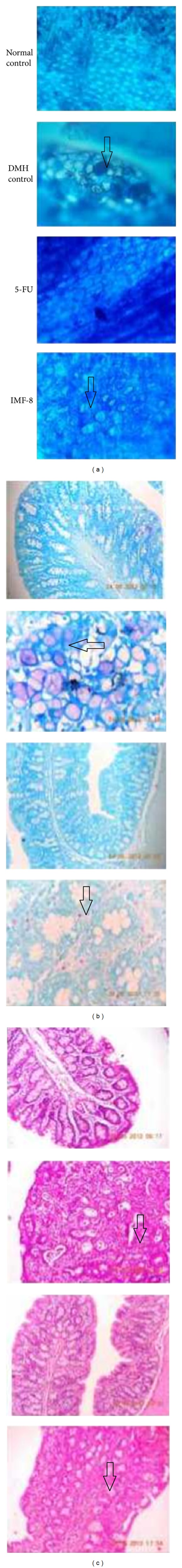
Histopathological images of the rat colons in normal control, DMH control, 5-FU, and IMF-8 treated groups. (a) Methylene blue (10x), (b) methylene blue (40x), and (c) haematoxylin-eosin stain (40x). Arrows indicate aberrant crypt foci. Normal control and 5-FU groups showed no signs of crypt dysplasia.

In normal control, colon showed no signs of crypt abscess and dysplasia. In DMH control group, colonic mucosa showed crypt abscess, aberrant crypt foci, and nuclear enlargement with adenocarcinoma. Intense methylene blue stained ACF is shown by arrow. Treatment with 5-FU and IMF-8 showed no signs of mucosal crypt abscess and exhibited a few ACFs (Figure 3).

## 4. Discussion

Flavonoids are proven to be of potential antiproliferative, anti-inflammatory, apoptotic, and anticancer activity with diverse cell systems [21]. Alvocidib (Flavopiridol) belongs to a family of flavonoids and is a cyclin-dependent kinase inhibitor under clinical development for the treatment of chronic lymphocytic leukemia and gastric cancer [22].

Fifteen of the 55 flavone derivatives were significantly active against at least one of these cell cultures [23]. The first step in the development of a new drug as a potential anticancer agent is the evaluation of its cytotoxic potential in cancer cell lines. In our study we evaluated a detailed anticancer potential of iminoflavones in HCT cancer cell line* in vitro* and in a suitable animal model* in vivo*. In this direction, four iminoflavones were screened for their cytotoxic potential against HCT cancer cell line using two different assays.

The specific way in which a cell reacts to its environment varies. It varies according to the set of receptor proteins the cell possesses, which determines the particular subset of signals it can respond to, and it varies according to the intracellular machinery by which cell integrates and interprets the signals it receives. Hence, different type of cells may respond differently to the same compound. Results obtained from these two basic assays demonstrated that IMF-8 was highly active against HCT-116. Next, we raised the question whether IMF-8 mediated cytotoxicity in the cancer cells is owing to necrosis and/or apoptosis which are key cell death processes. As a control, untreated cells were cultured in complete media and stained with AO/EB. The data showed that IMF-8 induced apoptosis after 24 h incubation. The percentage of apoptotic cells after treatment was increased significantly compared to control in HCT-116 cells. AI was also significantly (*P* < 0.01) increased in treatment groups when compared to untreated group. This is suggestive of the iminoflavone's capability of inducing apoptosis, especially those cells which are in the final stages of apoptosis, significantly. Since with Hoechst stain there was a significant rise in apoptosis with IMF-8, it shows that the flavone could possibly bind to DNA and would have interfered with DNA replication during cell division.

Colon carcinogenesis is a multistage process which involves initiation, promotion, and progression phases. Thus, the multistage sequence of events has many phases for prevention and intervention. Chronic inflammation is a well-recognized risk factor for development of human cancer. In the present study, DMH control animals showed initiation of ACF, promotion into adenoma, and the significant increase in inflammatory mediator (TNF-*α* and IL-6) levels.

DMH is a chemical carcinogen known to cause colon cancer with a reproducible experimental* in vivo* system for studying “sporadic” (nonfamilial) forms of colon carcinoma [24]. Metabolism of DMH leads to the formation of AOM, MAM, and methyl diazonium ions which involves alkylation of colonic mucosal DNA. The primary metabolite of DMH, that is, AOM, which is responsible for methylation at the O-6 position of guanine, occurs within 6 to 12 h of DMH injection.

ACFs are considered to be an earliest hallmark of colon carcinogenesis. They are the focal lesions of colonic mucosa consisting of several enlarged/aberrant/elliptical shape crypts which can be differentiated from normal crypts [25]. Considering this fact, we confirmed the induction of colon cancer and grouped the animals by evaluating the colonic ACFs with methylene blue staining.

The mutations in k-ras, hypo-Met-DNA in ACFs lead to the development of small and large adenoma, which will further develop into adenocarcinoma by the mismatch repair and p53 mutation [26]. We observed the initial lesions of ACF and development of small adenoma in DMH control group, whereas only ACFs and polyps, but not adenomas, were noted in the rest of the groups. Also some studies showed that DMH administration had increased levels of TNF-*α* and IL-6 and decreased endogenous antioxidant enzyme levels [27].

The increases in ACF count and number of polyps were used to evaluate the extent of damage to the colon caused by DMH. DMH treatment showed increase in ACF count with adenoma. The standard drug 5FU and IMF-8 showed better anticancer efficacy in terms of maintaining lower number of ACFs, polyps; lower levels of TNF-*α*, IL-6; significantly higher levels of catalase enzymes, and to some extent higher levels of GSH enzyme as well. IMF-8 showed comparable efficacy with respect to the standard 5-FU in this model with an additional anti-inflammatory activity and by enhancing endogenous antioxidant enzyme levels. Histopathological studies showed mucosal crypt abscess, huge number of aberrant crypt foci, and nuclear enlargement with adenocarcinoma in only DMH treated group, whereas there was less number of aberrant crypt foci with no signs of mucosal crypt abscess in 5-FU and IMF-8 treated groups.

In view of the chemical stability of compound, IMF-8, a “dimethylamino” substituted iminoflavone, has comparatively better electron donating property (a lone pair of electrons) which confers the structure higher stability. Based on chemical stability,* in vitro* anticancer activity, reduced ACF count, improved endogenous antioxidant enzymes, decreased inflammatory mediator levels, and histopathological findings, we conclude that IMF-8 could be a promising flavonoid for combating against colon carcinogenesis.

The exact mechanism of IMF-8 was difficult to establish since it had multifaceted actions. However, it is known that a majority of naturally occurring flavones show tumor growth inhibition in cancer cells through tyrosine kinase receptor inhibition [28]. But we speculate that the investigational iminoflavone IMF-8 mediated cytotoxicity in cancer cells could be due to apoptosis.

## Figures and Tables

**Table 1:** Preliminary cytotoxicity assessment in various cell lines.

Treatment	IC_50_ in *μ*M (mean ± SEM)			
	HCT-116	MCF-7	MDA-MB-231	EAC
Doxorubicin	2.88 ± 0.02	4.43 ± 0.34	1.77 ± 0.1	25.05 ± 0.9
IMF-2	105.2 ± 0.9	ND	ND	ND
IMF-4	132.1 ± 1.2	ND	ND	ND
IMF-5	41.79 ± 0.67	42.53 ± 0.78	59.13 ± 0.76	110.2 ± 0.21
IMF-8	2.78 ± 0.10	13.51 ± 0.13	6.38 ± 0.24	36.6 ± 3.2

Values are reported as mean ± SEM of three readings in triplicate; ND: not determined.

HCT-116, MCF-7, and MDA-MB-231 by SRB assay (exposure: 48 h).

EAC by Trypan blue exclusion assay (exposure: 4 h).

**Table 2:** Effect on the apoptotic indices by AO/EB and Hoechst 33342 staining.

Apoptotic indices (mean ± SEM)		
Groups	AO/EB staining	Hoechst 33342 staining
Control	3.7 ± 1.2	6.7 ± 0.88
Doxorubicin	46 ± 1.73^a^	38.7 ± 2.40^a^
IMF-8	33.3 ± 3.76^a^	34.7 ± 2.96^a^

Values are reported as mean ± SEM of three readings in triplicate.

^a^ *P* < 0.01 when compared to the control group.

**Table 3:** Effect on ACF incidence, number of ACFs, and size of polyps (adenomas) in DMH-induced colon cancer in rats.

Groups	ACF incidence (%)	Polyps (adenomas)				
		Number of ACFs/cm^2^ (mean ± SEM)	Small (0.1–1 mm)	Medium (2–4 mm)	Large (5–8 mm)	Total count
Control	0	0	0	0	0	0
DMH	100.0	16.85 ± 1.5^a^	4	5	4	13
5-FU	100.0	11.6 ± 1.0^a^	3	3	1	7
IMF-8	100.0	12.5 ± 1.6^a^	4	5	0	8

Values are reported as mean ± SEM of three readings in triplicate.

^a^ *P* < 0.01 when compared to the control group.
